# Simple citrate anticoagulation protocol for low flux haemodialysis

**DOI:** 10.1186/s12882-018-0811-y

**Published:** 2018-01-19

**Authors:** Eng Kuang Lim, Ying-ying T Seow, Shun E Chen, Gao Yang, Min Er Liaw, Shimi Isaac

**Affiliations:** Division of Renal Medicine, Khoo Teck Puat Hospital, Singapore, 768828 Singapore

**Keywords:** Anticoagulation, Citrate, Haemodialysis

## Abstract

**Background:**

For patients unable to receive heparin anticoagulation during haemodialysis, saline flushes to reduce circuit clotting are often the norm. Regional citrate anticoagulation (RCA) although effective is not used by many centres including in Singapore. We wanted to demonstrate the superiority and safety of a simple regional citrate anticoagulation regime, compared to saline flushes, for heparin-free low flux haemodialysis.

**Methods:**

This is a prospective, open label, cross over study on 25 sequential haemodialysis sessions for inpatients receiving heparin-free haemodialysis. Patients were allocated either to regional citrate anticoagulation or standard heparin free haemodialysis and subsequently cross over to the alternate method. RCA was carried out using a protocol derived from previous studies. Assessment of anticoagulation was performed using visual inspection of clot formation in dialysis circuits and post-filter ionized calcium (iCa2+) using point-of-care Ionized calcium device at stipulated intervals. Intravenous Calcium gluconate replacement was given to patients receiving citrate adjusting the rate according to pre-filter iCa2+. Laboratory analyses of electrolytes were also assessed at the start and end of the RCA sessions.

**Results:**

There were no clots in the RCA arm, with 79% (*n* = 19) in the saline flush arm having some clot, including 1 clotted circuit. Post-filter iCa2+ at various time points were within acceptable range.

Electrolyte readings in the RCA group were all within normal limits except for 4 cases of total Calcium:iCa2+ ratio > 2.5.

**Conclusion:**

RCA is confirmed to be superior to saline flushes for circuit patency. We have a simple and safe protocol that can be followed for low flux haemodialysis.

The study was approved by Singapore National Health Group domain-specific ethnical committee. NHG DSRB reference number 2014/01037.

**Trial registration:**

Trial registration number: ISRCTN69952745 (registration date 8/11/17).

## Background

Conventional haemodialysis requires anticoagulation to prevent clotting in the extracorporeal circuit. The most frequently used anticoagulants for chronic intermittent haemodialysis are unfractionated heparin or low-molecular-weight heparin. However, for patients with high bleeding risk such as post-operative patients, or with other contraindication for use of heparin as in Heparin-induced thrombocytopenia, heparin cannot be used. Current standard practice for this group of patients is to perform heparin-free haemodialysis with frequent saline flushes of the dialysis circuit. However, this method increases nursing time and effort and is of doubtful efficacy.

Regional citrate anticoagulation (RCA) was introduced in 1961 and is well-proven to be safe and effective in delivering adequate anticoagulation in extracorporeal circuits [1]. The anticoagulation effect of citrate is from chelation of ionized calcium which is an essential cofactor of the coagulation process. The benefit of this method is that anticoagulation is limited to the dialysis circuit. Serious complications include systemic hypocalcaemia and metabolic alkalosis, which if severe can be life threatening [2]. Protocols requiring customized dialysate solutions, complicated citrate/calcium infusion titration have impeded wide utility of citrate anticoagulation. The unavailability of calcium-free dialysate in Singapore further limited the development of this procedure locally.

We wanted to establish a simple protocol for regional citrate anticoagulation using a constant citrate infusion rate and commercially available calcium free dialysate, with titrated calcium replacement based on patient’s ionized calcium levels.

## Methods

### Study design and study population

This was a prospective, open label, cross over study. Hospital inpatients scheduled for at least 2 heparin-free hemodialysis sessions between August 2015-Jul 2016 were recruited. Inclusion criteria: Older than 18 years, Haemoglobin >7.0 g/dL, haemodynamically stable with a reasonably functioning vascular access. Both chronic hemodialysis patients and acute kidney injury patients were included. Exclusion criteria: decompensated liver disease, received blood products within last 24 h/required blood products during the dialysis session, hemostatic disorders or deranged clotting or known allergy to citrate products. Patients received consecutive dialysis sessions alternating control (saline flushes) with RCA, either arm could be administered first. The same blood and dialysate flow as well as access were used in both arms. Any single patient could be recruited for a maximum of 2 cross over dialysis sessions.

### Protocol

All dialysis treatments utilized a Gambro AK96 machine and Gambro Polyflux® 14 L or 17 L dialysers (institutional practice at the time of the study). Dialyser choice, blood flow rate (QB) of 150-250 ml/min and dialysate flow rate (QD) of 300-500 ml/min, and ultrafiltration volume were as ordered by primary nephrologist. All sessions were approximately 4 h long (minimum 3 h 45 min).

For the control arm, Gambro SoftPac™ C298 dialysate was used with sodium set at 138mmMand bicarbonate set at 32 mM. 100 ml saline flushes were given per 30 min, extra flushes were allowed as per nurses’s discretion according to standard practice of monitoring for clots in the circuit. Total saline flushing volume was recorded. Dialysis circuit priming was with saline or saline with heparin as ordered. For the RCA arm, Calcium-free dialysate (SW 415A, B. Braun Avitum AG, Germany) was used with dialysate sodium set at 136 mM and bicarbonate at 30 mM. Both dialysates contained glucose 1 g/L Table 1. Dialysis circuit was primed with saline only.

**Table 1:** Dialysate composition

	RCA	saline
Acidification	Acetate 3 mM	Acetate 3 mM
Sodium (mM)	136	138
Magnesium (mM)	0.5	0.5
Calcium (mM)	0	1.25

**Fig. 1:**
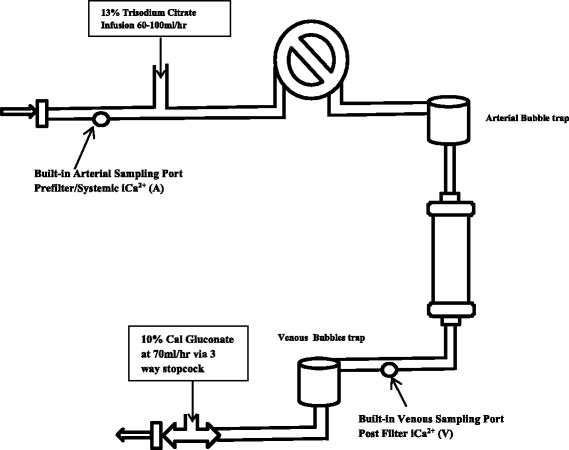
Circuit with sampling points

For RCA, we used a protocol derived from various studies [3–5]. Fig. 1 shows points of infusion and iCa2+ testing.

Trisodium Citrate 13% or 500 mM (Dirinco BV, Netherland) was infused at a fixed rate pre-filter (into the “heparin” line) according to blood flow (QB) as shown in Table 2.

**Table 2:** Trisodium Citrate 13% Infusion

Blood flow	Trisodium citrate 13%
150 ml/min	60 ml/h
200 ml/min	80 ml/h
250 ml/min	100 ml/h

Calcium Gluconate 10% (2.2 mmol Ca2+ in 10 ml vial) was infused post filter (using a 3-way connector into the “venous” return line). Infusion rate was started at 70 ml/h. Pre-filter iCa2+ level was sampled from port A, Fig. 1. This means it is taken upstream from the citrate infusion point hence reflecting systemic iCa2+. Calcium infusion rate is then adjusted accordingly as shown in Table 3.

**Table 3:** Calcium Gluconate 10% Infusion

Point A: Systemic iCa2+ Level	Calcium Gluconate Infusion
<0.65 mM	Notify doctor. Terminate citrate infusion and change to heparin free dialysis. Give 40 ml bolus over 30 mins
0.65–0.74 mM	Give 20 ml bolus over 30 mins and increase infusion by 20 ml/h
0.75–0.9 mM	Increase infusion by 20 ml/h
0.91–1.2 mM	Infuse at 70 ml/h
>1.2 mM	Hold infusion for 30 mins and recheck

Post-filter iCa2+ taken post-filter at point V, Fig. 1, was kept between 0.25–0.35 mM. Any reading outside this range, the doctor would be informed and adjustments made according to doctor’s discretion.

### Data collection, sample processing

Arterial, venous and transmembrane pressures in the extracorporeal system and blood flow and ultrafiltrate rates were recorded half hourly/additionally as required by the attending nurse. All technical problem and adverse events were specifically noted as well. At the end of the dialysis session, arterial and venous drip chambers and the filter were inspected by the nurse for visible signs of coagulation. A score of 1 (no clots), 2 (small clots), 3 (large clots) and 4 (complete clotting of circuit) was applied. Post-filter iCa2+ (V) were tested at time 30, 60, 120 and 240 (just before ending) minutes. Pre-filter iCa2+ levels (A) were measured at 0, 30, 60, 120 and 240 min. Determination of iCa2+ and blood gas analysis at all time points were drawn into heparinized syringes and processed at the bedside using OPTI CCA TS ABG Analyser (OPTI Medical Systems Inc., USA). In addition, blood samples were sent to the laboratory at time zero and 240 min (just before ending).

The nurse performed half hourly assessments of the patient for symptoms of hypcalcaemia (peripheral tingling, peri-oral dyasaesthesia, tetany or arrhythmia) or hypercalcaemia (sensation of warmth, nausea/vomiting, mental confusion). Signs or symptoms of citrate toxicity such as hypotension were carefully monitored for [2].

### Statistical analysis

Normally distributed data are expressed as mean ± standard deviation with differences tested using paired t test. Non-normally distributed data are expressed as median with range. Statistical significance was set at a two-tailed *p* value of <0.05.

## Results

### Study population

A total of 25 RCA and 24 saline flush dialysis sessions were performed. 22 patients were recruited with 3 recruited twice. 1 patient underwent only RCA dialysis only and had no control arm. All were chronic haemodialysis patients (Table 4).

**Table 4:** Baseline demographics median (range/%)

	RCA (*n* = 25)	Control (*n* = 24)
Male	19 (76%)	18 (75%)
Age (years)	57.0 (24–75)	57.5 (24–75)
Weight (kg)	70.0 (42–150)	69.5 (42–150)
Slow Low Efficiency Dialysis (SLED) (QB150ml/min QD 300 ml/min)	5 (20%)	5 (21%)
Vascular Access (AVF)	7 (28%)	7 (29%)
Haemoglobin (g/dL)	9.2 (7.2–14.1)	9.2 (7.2–14.1)

### Thrombosis and circuit loss

There were no clots within the RCA arm (0/25), while for the saline flush arm, 79% (19/24) had some clot, 1 patient actually requiring repriming of the entire circuit.

### Anticoagulation

**Fig. 2:**
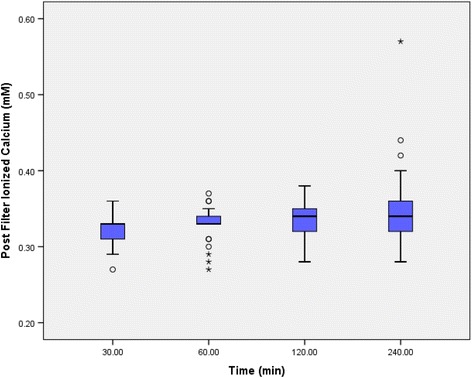
Post-filter iCa2+ (V)

All post-filter iCa2+ readings were within target range of 0.25–0.35 mM between 30 and 120 min without need for discretionary adjustments by the doctors (Fig. 2). Only 3 readings had level > 0.4 mM and these were all at 240 min. This suggested that the protocol achieved desired anticoagulation throughout the 4 h in almost all cases [2, 6].

### Electrolytes and calcium for RCA sessions

**Fig. 3:**
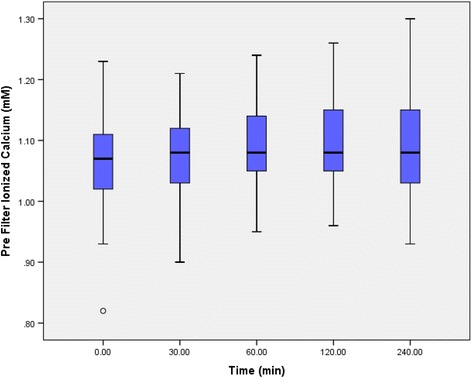
Pre-filter iCa2+ (A)

pH and bicarbonate (HCO3) was higher post-dialysis as expected (Table 5). Although total Calcium levels were increased significantly post-dialysis, change in iCa2+ was insignificant (Fig. 3 and Table 5). Magnesium levels were not significantly altered (Table 5).

**Table 5:** Electrolyte changes during RCA sessions (mean 95% confidence interval)

	0 min	240 min	Mean change (95% Confidence Interval)	*p*
Bicarbonate (23-29 mM)	20.9 (±2.8)	24.6 (±1.9)	+3.8 (2.9–4.7)	<0.001
pH (7.200–7.600)	7.38 (±0.041)	7.43 (±0.034)	+0.05 (0.03–0.06)	<0.001
Magnesium (0.6–1.1 mM)	0.95 (±0.17)	0.88 (±0.09)	−0.07 (0.02–0.11)	0.431
Total Calcium (2.15–2.50 mM)	2.12 (±0.13)	2.54 (±0.28)	+0.42 (0.30–0.53)	<0.001
iCa2 + ((1.13–1.32 mM))	1.07 (±0.09)	1.09 (±0.09)	+0.03 (0.02–0.07)	0.248

### Complications and adverse events

No patient in the RCA arm experienced any symptoms of hypo nor hypercalcaemia. There was 1 patient who experienced asymptomatic intradialytic hypotension during both the RCA and heparin-free arm. During both procedures, ultrafiltrate volume was adjusted and the patient stabilized. During the RCA arm, it was not felt to be due to citrate toxicity and hence RCA dialysis was continued.

There were 4 patients with total Calcium: iCa ratio > 2.5 (TCa: iCa2+). This was not related to age, gender, weight, QB/weight ratio, Haemoglobin (data not shown). All received normal haemodialysis and not SLED.

### Technical problems encountered

1. Median volume of citrate and calcium infused was 700 ml, this had to be factored into the final ultrafiltrate volume which was similar to using saline flushes, limiting net ultrafiltrate achievable within 4 h.
2. While only 1 bag (1000 ml) of 13% citrate was used per patient but only a median volume of 400 ml was required. Conversely, a median number of 30 vials of 10 ml calcium gluconate were required per patient. We were unable to obtain larger volume calcium gluconate vials, and cost consideration made us use calcium gluconate rather than chloride.

## Discussion

RCA is clearly superior to saline flushing for avoiding thrombosis in the dialysis circuit in our study, and has been shown to be superior even to low dose heparin by others [7, 8]. With our current protocol, electrolyte abnormalities such as alkalosis, hypocalcaemia and hypomagnesemia that have been associated with RCA did not occur [2].

We included 1 patient who only underwent RCA with no control arm since he had a TCa:iCa2+ > 2.5. There were 4 cases of high TCa: iCa >2.5 suggesting citrate accumulation. Measurement of serum citrate levels are not performed routinely and total Cal: iCa2 + ratios are usually accepted to correlate with serum citrate levels [9, 10].

Low flux dialyzers would be less efficient at citrate removal (<60% versus >80% with high flux [3, 4, 11]. Hence there would be more dependence on patient’s metabolism to breakdown citrate. If the patient’s mitochondrial Kreb’s cycle pathway is abnormal, or tissues with high mitochondria content is abnormal eg liver dysfunction, decreased microcirculation to skeletal muscle eg in sepsis, the elderly or women with lower muscle bulk may also have lower citrate metabolism. Volume of distribution for citrate is mainly determined by state of hydration/water compartment so for the very oedematous/dehydrated or critically ill with impaired microcirculation citrate clearance may not be so predictable [2]. Due to the small sample size, the reason(s) for presumed citrate accumulation in these 4 cases are hence unclear and there was no clear association found in our study for some of these factors.

From our study, the distribution of post-filter iCa2+ readings at 1 h, although all within desirable range, but had an uneven spread. With adjustments at that stage per protocol, the 120 min readings fall within the 95% CI. Hence In our next large scale protocol, our intent is to do just baseline (pre-filter) and 1 h (pre and post-filter) iCa measurements. Additional tests are required only if 1 h reading is outside desirable range.

### Limitations of the study

Numbers were small, but as mentioned this was a pilot to test out a simple protocol prior to a large scale study. Also because of the numbers, we could not analyse if there was actually any association between high TCa: iCa2+ ratios and various factors known to result in citrate accumulation. We used the available POCT iCa2+ testing equipment that was available. Some studies suggest that certain POCT iCa2+ may not be accurate [12].

## Conclusion

Our study shows that RCA is a safe and effective mode of anticoagulation and should be considered as a preferred method in heparin-free hemodialysis. Once familiar with circuit setup, the simple protocol was easy for the nurses to follow. Use of low flux dialysers may increase the risk of citrate accumulation though this may not be of major clinical significance in most patients. Although not formally assessed but feedback from all the nurses involved in the study suggested their confidence in it and especially in the clearly superior result compared to saline flushing, there have been requests by them to use it after project termination.

## Abbreviations

iCa2+: Ionized Calcium
QB: Blood flow
RCA: Regional citrate anticoagulation
SLED: Slow Low Efficiency Dialysis
TCa: Total Calcium
TCa: iCa2+: Total Calcium: ionized Calcium ratio
